# PP08 Adaptive Curriculum In Health Technology Assessment: The Experience Of Cooperation Pedagogy In A Professional Master’s Program

**DOI:** 10.1017/S0266462325102286

**Published:** 2025-12-29

**Authors:** Flavia Tavares Silva Elias, Erica Tatiane da Silva, Fabio Otuzi Brotto, Luiza Siqueira do Prado

## Abstract

**Introduction:**

Emerging regions require equitable training processes to implement health technology assessment (HTA) effectively. This work presents the initial adaptive curriculum designed for a professional master’s degree in HTA, tailored to the regional context in Brazil, particularly for areas with evolving infrastructure. The objective was to foster HTA expertise by enhancing methodological skills, technical knowledge, and collaborative abilities among students.

**Methods:**

The curricular adaptation was developed through collaboration between students and course coordinators, utilizing a cooperative methodology grounded in the first four stages of the Cooperation Pedagogy framework.

**Results:**

The class consisted of 20 students: 75 percent were women, 60 percent from the Central-West region, 30 percent from the Northeast, and 10 percent from the North. Ten students worked in HTA units in hospitals and two worked in legal units. Students’ topics of interest included vaccination in remote areas, judicialization, post-incorporation assessments, and budget impact analyses. The results of the four stages were: (i) a coexistence contract developed based on students’ expectations; (ii) a “World Café” dynamic that facilitated the creation of “hot questions”; (iii) the curricular adaptation integrating all relevant subjects; and (iv) the affective environment fostering mutual learning and collaboration.

**Conclusions:**

The next steps involve co-creating solutions to the students’ hot questions, which were: (i) Does decision-making overturn an HTA report?; (ii) Is the HTA language understandable to stakeholders?; (iii) How to raise awareness among judges?; (iv) How to stimulate the creation of HTA units in emerging regions?; and (v) What is the future of HTA?

